# The influence of tumor necrosis factor microsatellite polymorphisms on patient survival following hematopoietic stem cell transplantation

**DOI:** 10.3325/cmj.2012.53.24

**Published:** 2012-02

**Authors:** Katarina Štingl, Renata Žunec, Ranka Serventi-Seiwerth, Boris Labar, Zorana Grubić

**Affiliations:** 1 Clinical Department for Transfusion Medicine and Transplantation Biology, University Hospital Center Zagreb, Zagreb, Croatia; 2 Department of Hematology, Internal Clinic, University Hospital Center Zagreb, Zagreb, Croatia

## Abstract

**Aim:**

To investigate the influence of tumor necrosis factor (TNF) microsatellite polymorphisms on patient survival following hematopoietic stem cell transplantation.

**Methods:**

We analyzed TNFa, TNFb, and TNFd microsatellites among 100 patients who underwent allogeneic hematopoietic stem cell transplantation from a human leukocyte antigen (HLA)-identical sibling donor at the Internal Clinic of the University Hospital Center Zagreb in the period 2001-2009. The analysis was performed using polymerase chain reaction amplification and electrophoresis on a polyacrylamide gel in an automated sequencer.

**Results:**

There was no significant difference in patient survival with respect to the allele length at a given microsatellite. However, a significantly lower survival rate was noticed among patients who were positive for TNFa8 allele (*P* < 0.001) and a significantly higher survival rate among those who were positive for TNFa10 allele (*P* = 0.0220).

**Conclusion:**

These results for the first time suggest an influence of TNFa microsatellite on patient survival following HSCT and indicate a need for further studies of this microsatellite.

Hematopoietic stem cell transplantation (HSCT) is considered to be the most effective treatment for numerous diseases and disorders of hematological system despite its considerable side-effects and treatment-induced mortality. One of the most common complications of this procedure is acute graft vs host disease (GvHD), which can occur even in the cases when transplantation is performed from a human leukocyte antigen (HLA)-identical sibling donor and which affects up to 50% of the patients (1). GvHD is a consequence of tissue damage and subsequent bacterial infiltration due to the conditioning of patients prior to the HSCT. The resulting release of the proinflammatory cytokines then triggers the activation of donor's T cells, which in turn leads to tissue damage of target organs and a possible multi-organ failure. The major role in this, so called cytokine storm, which constitutes both the induction and effector phase of the GvHD, is played by tumor necrosis factor α (TNFα) cytokine (2). The level of TNFα during the conditioning period has been associated with the severity of the acute GvHD (3).

The role of polymorphisms located within the TNF gene cluster region in the etiology of various diseases that have shown an association with the HLA system has been extensively studied. A large number of single nucleotide polymorphisms and 6 microsatellites have been described in the TNF region to date. The first five microsatellites are referred to as TNFa, TNFb, TNFc, TNFd, and TNFe (4). The sixth microsatellite (TNFf) was described later (5). TNFc, TNFd, and TNFe are (GA)n repeats, whereas TNFa and TNFf are (GT)n and (CA)n repeats, respectively. The only exception of this dinucleotide repeat motif is the TNFb microsatellite, which is composed of mononucleotide tandem repeats (G/A)n. The most polymorphic TNF microsatellite is TNFa with 14 alleles, while 7 alleles have been reported so far for TNFb and TNFd loci each (4). The role of these microsatellites was implicated in the development of diseases such as rheumatoid arthritis, multiple sclerosis, or type 1 diabetes (6-8). However, in the transplantation field, the most intriguing aspect of these polymorphisms is that, according to several studies (9,10), the level of the TNFα secretion is influenced by the presence of some alleles at the TNF microsatellite loci. Taking into account the importance of this cytokine in the induction of GvHD, the information about the potential of the patient’s immune cells to produce it is of great value in the evaluation of risk for GvHD occurrence and prediction of HSCT outcome. The analysis of these microsatellites could therefore be applied in the detection of those patients who are at higher risk of developing GvHD after HSCT (11).

The aim of this study was to investigate the importance of selected TNF microsatellites in the patient survival following HSCT. The choice of the loci included in the analysis (TNFa, TNFb, and TNFd) was guided by the above mentioned study (11), as well as by the level of polymorphism detected for a given locus in the Croatian population (12).

## Materials and methods

The study included 100 patients who were part of the transplantation program at the Internal Clinic of the University Hospital Center Zagreb in the period 2001-2009, and received the transplant from an HLA-identical sibling donor. The criteria for the inclusion of a given patient into the study were the type of conditioning (myeloablative protocol) and donor (HLA-identical sibling). The following patients’ characteristics were noted: age, diagnosis, sex and sex-matching with the donor (Table 1). The control group consisted of 150 healthy individuals who were analyzed for TNF microsatellites in a previous study (12).

**Table 1:** Characteristics of patients (n = 100) who received the transplant from a human leukocyte antigen-identical sibling donor

Characteristic	No. of patients
Age, years, (median, range)	26 (1-53)
Diagnosis:	
acute myeloid leukemia	28
acute lymphocytic leukemia	25
chronic myeloid leukemia	8
chronic lymphocytic leukemia	2
non-Hodgkin’s lymphoma	7
aplastic anemia	13
myelodysplastic syndrome	5
other	12
Sex, male/female	54/46
Sex matching of patient and donor, matched/mismatched	50/50

DNA was isolated from the peripheral blood using the Nucleospin® Blood kit (Macherey-Nagel, Duren, Germany). All samples were analyzed for three TNF microsatellites (TNFa, TNFb, and TNFd). The analysis was performed using polymerase chain reaction (PCR) with specific, fluorescently labeled primers according to the protocol reported by Udalova (4). The PCR amplification was followed by electrophoresis on a 6% polyacrylamide gel in an automated sequencer ALFexpress (Amersham Pharmacia, Uppsala, Sweden). The calculation of the length of amplified fragments and allele assignation was performed using the AlleleLocator software (Amersham Pharmacia, Uppsala, Sweden). The alleles were designated as TNFa1-TNFa14, TNFb1-TNFb7, and TNFd1-TNFd7. The names of the alleles reflect the number of the basic motif repeats within the allele, eg, TNFa6 allele has 6 repeats of the GT sequence. TNFa alleles ranged from 97 bp to 123 bp, TNFb alleles from 124 bp to 130 bp, and TNFd alleles from 124 bp to 136 bp.

In order to evaluate the possible influence of the patient's TNF microsatellite allele length on the patient survival following HSCT, the participants were divided into three groups: individuals who carried alleles TNFa1-TNFa6 at the TNFa locus were included in group 1, those who carried alleles TNFa7-TNFa13 were included in group 3, and those who carried one short and one long allele were included in group 2. The same principle was applied for TNFb and TNFd loci with the first three alleles at both loci being classified as short alleles and the remaining four alleles as long alleles.

The allele frequencies were obtained by direct counting. In the cases when only one allele was detected, the person was considered homozygous for the microsatellite locus in question. Data were analyzed in contingency tables by Yates χ^2^ test with 0.050 level of statistical significance, and by Fisher exact test when there were fewer than five cases. *P* value was corrected by multiplication with the number of detected alleles (*P*corr). Kaplan-Meier analysis was performed in the estimation of patient survival. The study design was approved by the University Hospital Center’s ethics committee and is in accordance with the Helsinki Declaration of 1975.

## Results

**Figure 1:**
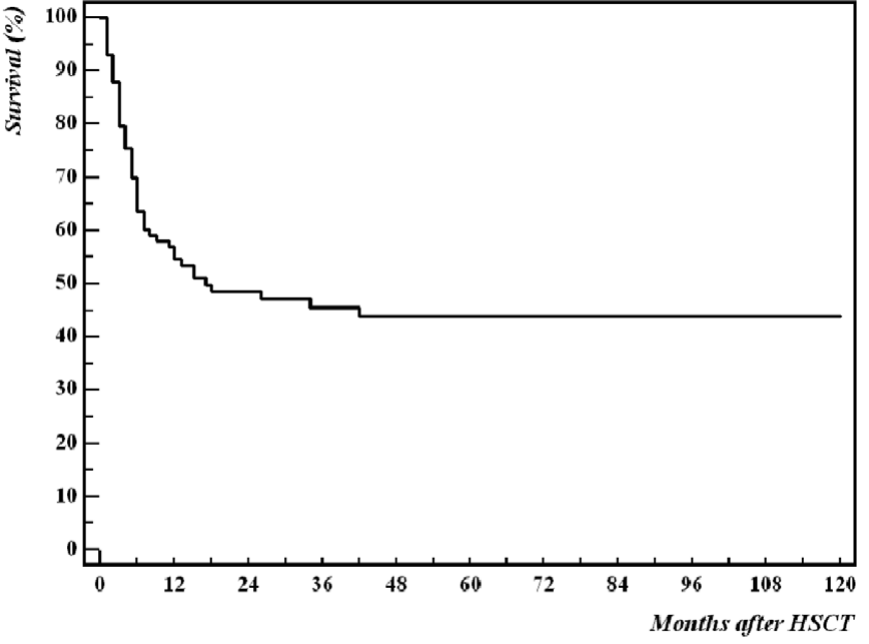
Total survival of all patients analyzed in the study (n = 100).

**Figure 2:**
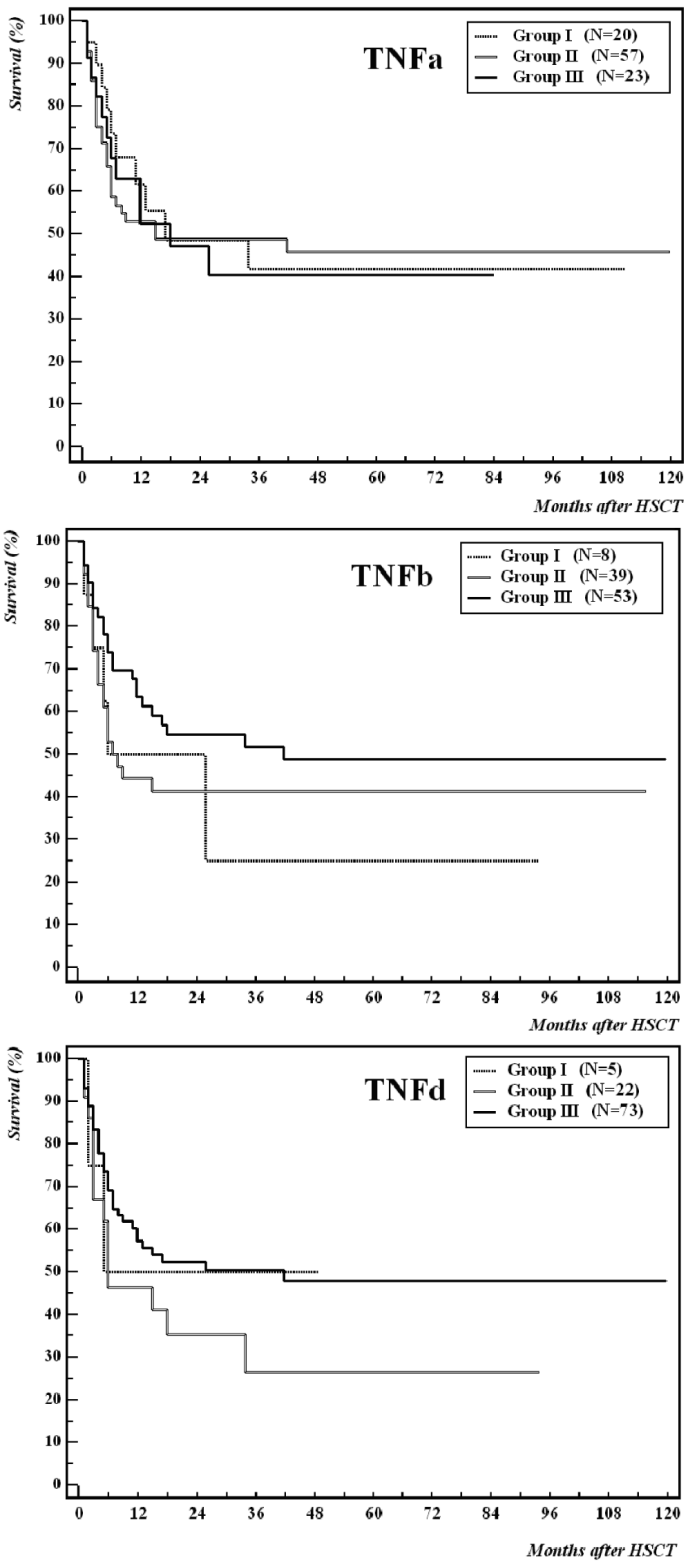
Patient survival according to tumor necrosis factor (TNF) microsatellites TNFa, TNFb, and TNFd allele length (N = 100). Group I – patients with two short alleles; Group II – patients with one short and one long allele; Group III – patients with two long alleles; short alleles: TNFa1-TNFa6, TNFb1-TNFb3, TNFd1-TNFd3; long alleles: TNFa7-TNFa13; TNFb4-TNFb7; TNFd4-TNFd7.

There was no significant difference between patients and control participants in the distribution of alleles at any of the tested microsatellites (Table 2). The survival curve was calculated for all patients included in the study (Figure 1). The second aim of the study was to evaluate the possible influence of TNF microsatellite allele length on patient survival following HSCT. For this purpose, patients were divided into three groups and compared according to patient survival (Figure 2). No significant difference was found for any of the three tested loci (Figure 2).

**Table 2:** The allele distribution at tumor necrosis factor (TNF) loci (TNFa, TNFb, and TNFd) among patients (n= 100) and control participants (n = 150)

TNF allele	No. (%) of patients	No. (%) of controls
TNFa1	3 (1.5)	2 (0.7)
TNFa2	32 (16.0)	50 (16.7)
TNFa3	7 (3.5)	5 (1.7)
TNFa4	13 (6.5)	16 (5.3)
TNFa5	14 (7.0)	25 (8.3)
TNFa6	30 (15.0)	39 (13.0)
TNFa7	13 (6.5)	26 (8.7)
TNFa8	4 (2.0)	-
TNFa9	5 (2.5)	12 (4.0)
TNFa10	51 (25.5)	71 (23.7)
TNFa11	18 (9.0)	34 (11.3)
TNFa12	-	-
TNFa13	10 (5.0)	19 (6.3)
TNFa14	-	1 (0.3)
TNFb1	20 (10.0)	42 (14.0)
TNFb2	1 (0.5)	9 (3.0)
TNFb3	34 (17.0)	39 (13.0)
TNFb4	83 (41.5)	140 (46.7)
TNFb5	57 (28.5)	63 (21.0)
TNFb6	-	3 (1.0)
TNFb7	5 (2.5)	4 (1.3)
TNFd1	4 (2.0)	7 (2.3)
TNFd2	14 (7.0)	17 (5.7)
TNFd3	15 (7.5)	7 (2.3)
TNFd4	107 (53.5)	171 (57.0)
TNFd5	39 (19.5)	64 (21.3)
TNFd6	17 (8.5)	28 (9.3)
TNFd7	4 (2.0)	6 (2.0)

**Figure 3:**
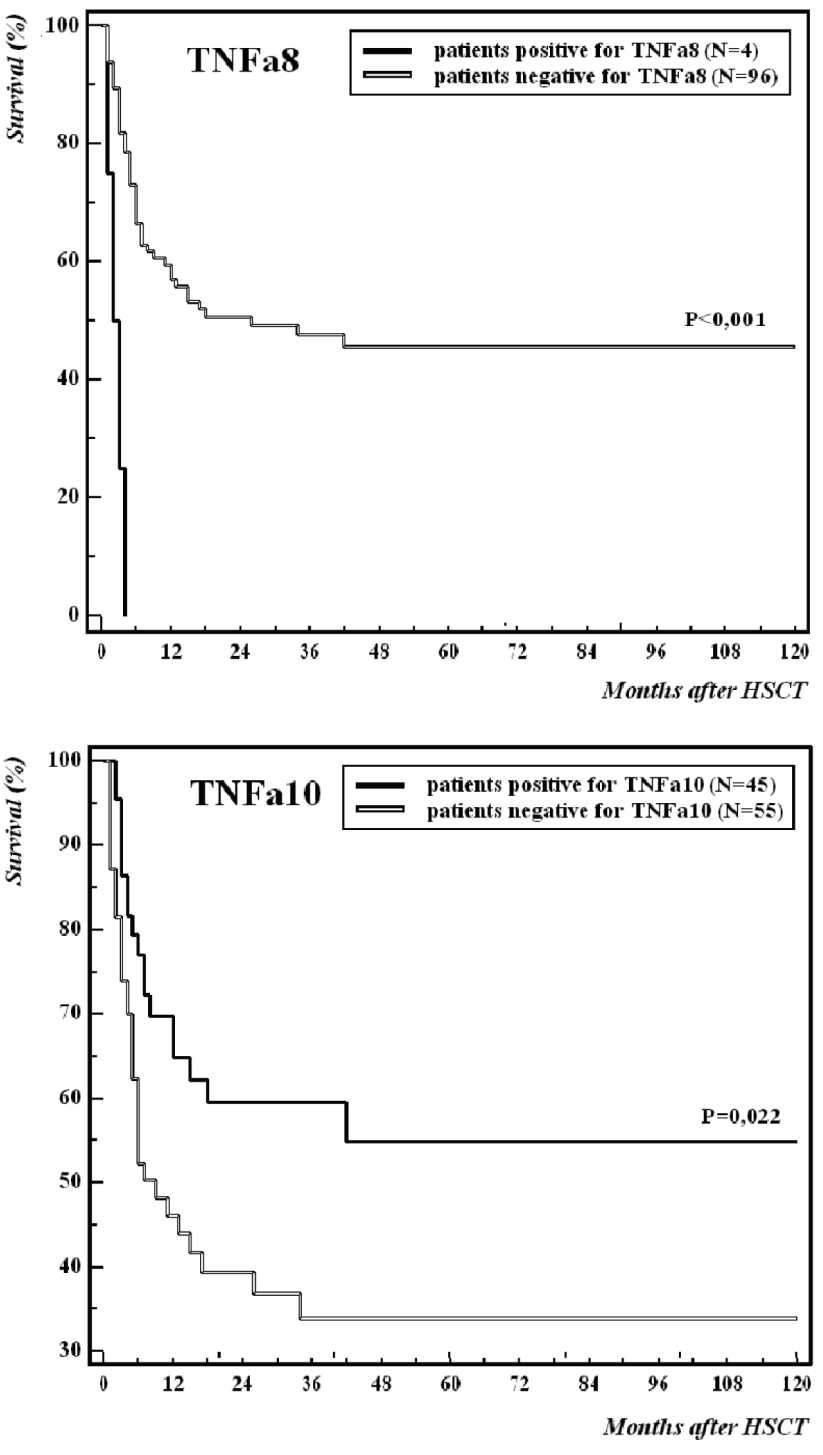
Patient survival according to the presence of tumor necrosis factor (TNF) alleles TNFa8 and TNFa10 (n = 100).

The final aim of this study was to compare the survival of patients according to the presence of an individual allele at the investigated TNF microsatellites. The alleles that showed an influence on the patient survival were TNFa8 and TNFa10 (Figure 3). Patients who carried TNFa8 allele (4.0%) had a significantly lower survival rate than patients who did not carry this allele (*P* < 0.001, *P*corr <0.001). Conversely, patients who carried TNFa10 (45.0%) had a significantly higher survival rate than patients who did not carry this allele (*P* = 0.022, *P*corr >0.050).

## Discussion

This study showed that TNFa microsatellite was a possible indicator of patient survival. The study was motivated by the work of Pociot et al (13), who demonstrated an association of polymorphisms located in the TNF region with the level of TNFα cytokine production. The hypothesis that launched further studies, including this one, is that the determination of TNF microsatellite alleles can have a prognostic value in the HSCT outcome and help in the selection of GvHD treatment and anticytokine therapy.

The first study about the influence of TNF region polymorphisms on the outcome of HSCT revealed an association of the recipient’s genotype TNFd3/TNFd3 with a higher occurrence of GvHD grade II and IV (11). Before that, TNFd3 had been shown to correlate with an increased production of TNFα (14). The finding that the TNFd3/TNFd3 genotype is associated with higher grades of GvHD was based on the analysis of only 49 patients, however, this result was later confirmed on a larger group (N = 144) of patients (15). A more recent study has demonstrated a significantly better survival of carriers of TNFd1/d2/d3 genotype than of carriers of TNFd3/d3 genotype, and TNFd4 and TNFd5 alleles (16). Finally, a Polish study has confirmed the association of TNFd3/d3 genotype with more severe forms of GvHD (17). Although TNFd3 allele has been emphasized as a risk factor by a majority of studies conducted so far, it is clear that a consensus has not yet been reached about the influence of TNF polymorphisms on the occurrence and severity of GvHD. This study did not indicate a correlation of the allele length at any of the tested loci and survival rates, although some difference was observed for TNFb and TNFd loci. However, while we could not compare our results for TNFb locus with other investigations, patients positive for TNFd1/d2/d3 genotype did not have a lower survival rate as was previously suggested (16). Also, as opposed to the studies that have highlighted TNFd3 allele as increased risk factor (13,15-17), this study showed an association of TNFa8 allele with a lower survival rate after HSCT, while the presence of TNFa10 allele correlated with a better prognosis.

In conclusion, this is the first study that indicated an influence of TNFa microsatellite on patient survival following HSCT. The results suggest that a more in-depth analysis of this polymorphism should be performed with respect to the severity of GvHD as well as other post-transplantation complications.
